# The Effect of Spatial Ability in Learning From Static and Dynamic Visualizations: A Moderation Analysis in 6-Year-Old Children

**DOI:** 10.3389/fpsyg.2021.583968

**Published:** 2021-06-18

**Authors:** Anis Ben Chikha, Aïmen Khacharem, Khaled Trabelsi, Nicola Luigi Bragazzi

**Affiliations:** ^1^Ksar-Saïd, Manouba University, ECOTIDI UR16ES10, Tunis, Tunisia; ^2^LIRTES (EA 7313), UFR SESS-STAPS, Paris-East Créteil University, Créteil, France; ^3^Research Laboratory: Education, Motricité, Sport et Santé, EM2S, LR19JS01, High Institute of Sport and Physical Education of Sfax, University of Sfax, Sfax, Tunisia; ^4^Laboratory for Industrial and Applied Mathematics, Department of Mathematics and Statistics, York University, Toronto, ON, Canada

**Keywords:** multimedia learning, spatial ability, young children, animation, cognitive abilities

## Abstract

Previous studies with adult human participants revealed mixed effects regarding the relation between spatial ability and visual instructions. In this study, we investigated this question in primary young children, and particularly we explored how young children with varying levels of spatial abilities integrate information from both static and dynamic visualizations. Children (*M* = 6.5 years) were instructed to rate their invested mental effort and reproduce the motor actions presented from static and dynamic 3D visualizations. The results indicated an interaction of spatial ability and type of visualization: high spatial ability children benefited particularly from the animation, while low spatial ability learners did not, confirming therefore the ability-as-enhancer hypothesis. The study suggests that an understanding of children spatial ability is essential to enhance learning from external visualizations.

## Introduction

Issues of spatial ability and learning achievement have been an underlying topic of psychological and educational discussions for many years (e.g., Presmeg, 1986; Wanzel et al., 2002; Unal et al., 2009). Concerning the spatial ability and its influence on learning from static and dynamic visualizations, numerous research has been conducted (e.g., Höffler, 2010; Höffler and Leutner, 2011; Nguyen et al., 2012; Berney et al., 2015; Castro-Alonso et al., 2018; de Koning et al., 2019; Kühl et al., 2018; Castro-Alonso et al., 2019a; Castro-Alonso et al., 2019b). However, studies investigating the effect of visualization type and spatial ability on children learning performances are lacking. Our study is, therefore, an attempt to directly examine this issue in the context of multimedia learning. Two principal research questions oriented this investigation: First, what external visualization will lead to the best understanding of a 3D game sequence in 6-year-old children? Second, does the efficiency of an external visualization depend upon children spatial ability?

Dynamic visualizations such as animations and videos can nowadays be easily integrated into a multitude of learning and training environments (Sherer and Shea, 2011; Kay, 2012; Khacharem et al., 2015a; Berney and Bétrancourt, 2016). It has been known that dynamic visualizations may facilitate learning as the learner can explicitly (and directly) perceive spatiotemporal changes in the depicted system/procedure. In the case of static visualizations, on the other hand, the learners have to mentally imagine spatiotemporal changes, which is assumed to be more challenging. Another argument suggests that the unequivocal depiction of a dynamic event through an animation can help the learner avoid misinterpretations of motion indicators used in static pictures, such as arrow symbols. Khacharem et al. (2015b) give the example of a diagram of play in which arrow symbols are used to depict players’ motion. Learners might incorrectly interpret/understand the significance and the amplitude of the depicted arrows. This may impose significant levels of cognitive load and lead to misunderstanding and consequently, to a deficient mental model (Lewalter, 2003; Khacharem et al., 2020). Additionally, the external depiction of a movement by a dynamic visualization is considered to be more entertaining and engaging than equivalent static visualizations, which may, in turn, lead to better learning results (e.g., Lepper and Malone, 1987; Rieber, 1991; Khacharem, 2017). Recently, some evidence has demonstrated that dynamic visualizations seem to be particularly efficient for teaching procedures/contents that are realistic, based on human movements, and involving procedural-motor knowledge (Höffler and Leutner, 2007).

However, it has been shown that the fleeting nature of dynamic visualizations generates transient information that can slow down their learning effectiveness (Ayres and Paas, 2007). The transient information effect is a loss of learning due to information disappearing before the learner has time to adequately process it or link it with new information (Sweller et al., 2011). Cognitive Load Theory (Sweller, 1994; Van Merrienboer and Sweller, 2005) suggests that the transitory nature of animations may impose extraneous cognitive load due to the temporal limits of working memory. When learning with dynamic visualizations, one frame is displayed at a time, and once the dynamic visualization has advanced beyond a given frame, that frame is no longer available to the learner. In this case, learners are required to process current information and integrate it with previous information at the same time. Such cognitive-perceptual processing may impose a higher cognitive load on working memory resources. Another argument suggests that animations may generate an illusion of understanding (Hegarty et al., 2003; Rebetez et al., 2010). An animation that provides the succession of steps and transformations over time from beginning to end (without interactivity) does not mobilize cognitive investment, but rather promotes passive rather than active learning.

Learning from external visualizations is considered to be an active process that is influenced by the prerequisites of the learner. One crucial factor mediating the effectiveness of such processes is learner spatial ability (e.g., Hegarty and Kriz, 2008; Schnotz and Rasch, 2008). Spatial ability refers to a group of cognitive functions and aptitudes that is crucial in manipulating and processing visuospatial information (Lajoie, 2008; Castro-Alonso and Atit, 2019). Spatial visualization ability is a measure of the ability to mentally rotate or fold objects and to imagine the changes in location and form due to this manipulation (e.g., Mayer and Sims, 1994). This ability varies significantly within humans; some individuals have a facility for transforming spatial information, while others find these processes very challenging (Caroll, 1993; Hegarty and Waller, 2005). Currently, two different hypotheses are employed to explain the relation between spatial abilities and presentations formats.

The ability-as-compensator hypothesis (Mayer and Sims, 1994; Höffler, 2010; Höffler and Leutner, 2011) posits that dynamic visualizations can assist low spatial ability learners by offering an explicit representation of temporal aspects of the system, thus reducing the need to mentally animating the static information. However, high spatial ability learners do not gain particular benefit from dynamic visualization because they are more cognitively equipped to generate an adequate mental representation of the depicted content regardless of the presentation format (Mayer, 2001). For example, Höffler and Leutner (2011) investigated the respective role of spatial ability and type of visualization (animation versus a series of static pictures) on learning of chemistry concepts. Spatial ability was measured using the Paper Folding test and the Card Rotation test (Ekstrom et al., 1976). The results indicated that low-spatial ability learners showed poor learning outcome when learning from static pictures while high-spatial learners did not. Conversely, when learning from animation, spatial ability did not moderate learning outcome as low and high spatial ability learners performed equally (Lee and Shin, 2012; Berney et al., 2015; Sanchez and Wiley, 2017).

On the other hand, the enhancer hypothesis (Hegarty and Sims, 1994; Hegarty, 2005; Huk, 2006; Höffler, 2010) claims that high spatial ability learners should uniquely benefit from the dynamic visualizations as they have enough cognitive capabilities left for mental model building of the content to-be-learned (Mayer, 2001; Huk, 2006). However, spatial ability learners experience an increase of unnecessary cognitive load while learning with static visualizations because their ability to mentally animate spatio-temporal information is limited (Hegarty and Sims, 1994; Hegarty, 2005; Huk, 2006; Keller et al., 2006; Höffler, 2010). Huk (2006) found that the incorporation of dynamic 3D models depicting a plant/animal cell enhance learning outcomes only in high spatial ability learners who are cognitively better ready to process dynamic visualizations since they have enough cognitive capacity left for building a coherent representation of the content to be learned. In contrast, low spatial ability learners are cognitively loaded by dynamic visualizations; therefore, they performed better with static visualizations.

A closer look at the aforementioned studies reveals that relatively little attention has been devoted to understanding the role of spatial abilities when learning from external visualizations in young children. Terlecki and Newcombe (2005) noted that young children have greater experience with modern multimedia technologies such as videos and computerized animations and, as a result, spatial ability could play an important role in learning processes. Previous research on spatial acquisition has indicated that mental paper folding emerges at 5.5 years of age and develops through early primary school (Harris et al., 2013). Similarly, it has been shown that enhancement in the ability to perform the object-based spatial transformations that necessitate spatial manipulation of mental image occurs from 5 years-old, although at a slower speed than adults (e.g., Frick et al., 2009; Funk et al., 2005; Kosslyn et al., 1990; Marmor, 1977; Crescentini et al., 2014). The purpose of this study was to explore the relative effects of spatial ability and type of visualization on children ability to learn a 3-D game sequence. This study employed the Mental Folding Test for Children (MFTC; Harris et al., 2013) and the Children’s Mental Transformation Task (CMTT; Levine et al., 1999; Ehrlich et al., 2006) in which adequate validity and reliability on assessing spatial visualization ability in children have been established. First, we hypothesized that watching an animation that explicitly depicted learning contents would result in better learning outcomes than watching a series of static pictures (Hypothesis 1). Second, based on the ability-as-compensator hypothesis, we expected that children with low spatial ability would principally benefit from animation, whereas children with high spatial would benefit equally from both static pictures and animation (Hypothesis 2).

## Materials and Methods

### Participants

A sample of 64 children (*M* = 6.5 years; SD = 0.23; 50% girls) in Grade 1 participated in this study. Children with intellectual disability, neurological disorder and/or uncorrectable hearing and/or visual impairment were excluded. They had not previously taken part in any similar research. The parents were required to consent to the inclusion of their child in the study and provide basic information on the child’s developmental history. The study was conducted according to the Declaration of Helsinki and fully approved by the Sfax University Ethics Committee (approval code CPP 0076/2017)” before the commencement of the study.

### Material Learning

**FIGURE 1:**
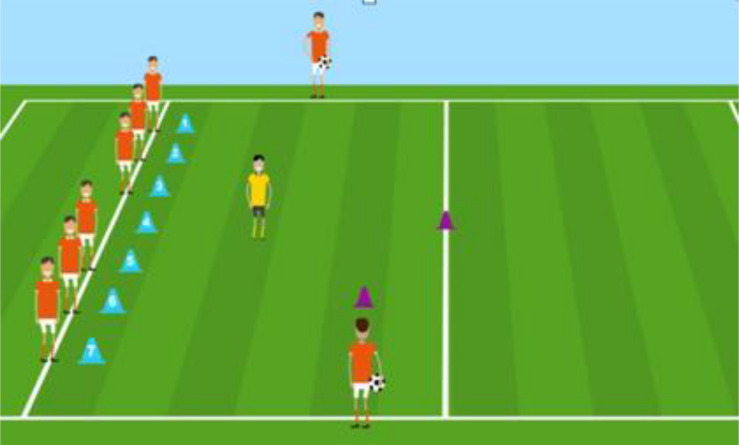
A screenshot from the 3D game animation.

A 3-D game sequence titled “the passing game” was designed and developed using Macromedia Flash MX Professional 2004. The game contained 10 players positioned as follows: seven players on the bottom line (attackers) and one player on each sideline (playmaker). It started with the teacher designing the number of an attacker (from 1 to 7) and ended with the designed attacker grounding the ball over the goal line. The game consisted of 11 steps. During each step, the attacker carried a set of actions: dribbling, hand passing, walking and accelerating. The game sequence of 47*sec* was presented via either an animation or a series of 12 static pictures representing the key moments of the sequence. Both versions were accompanied by the same verbal commentary. The learning and output stimuli were presented on a 17-inch LCD computer screen with a 1,280 × 1,024-pixel display. Figure 1 gives a screenshot from the 3D game sequence used in the study.

### Measures

#### Spatial Ability

**FIGURE 2:**
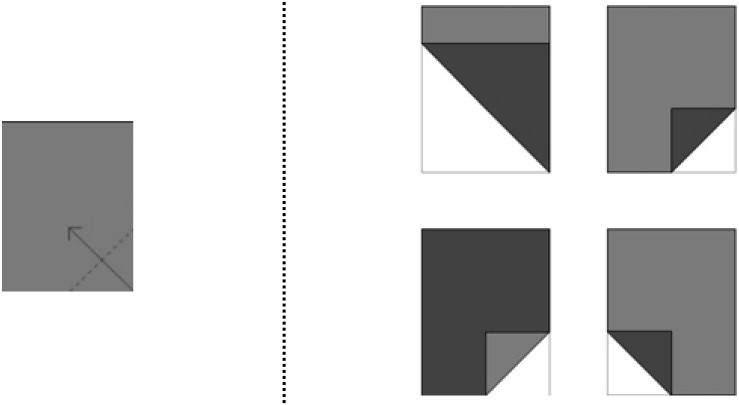
The Mental Folding Test for Children.

Children’s individual spatial ability was evaluated by two different tests. The MFTC (Harris et al., 2013) is a test developed for measuring the 4–7 years old children’s ability to fold 2D shapes in their mind. It is a multiple-choice test where both sides of the shapes are presented in different colors. Figure 2 shows one of the test items of the MTFC.

**FIGURE 3:**
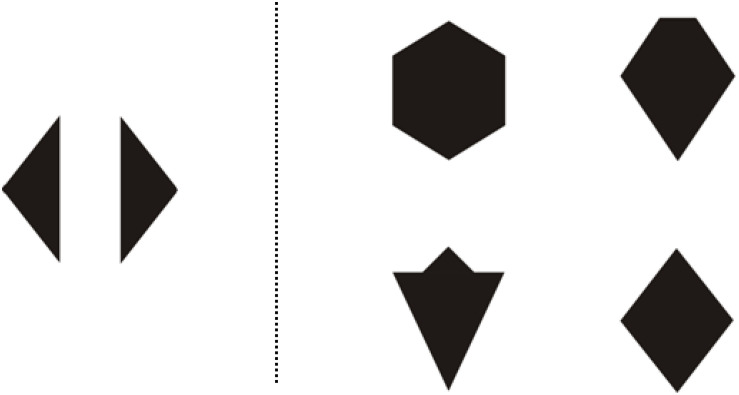
The Children’s Mental Transformation Task.

Another measure was the CMTT (Ehrlich et al., 2006; Levine et al., 1999) which consists of a multiple-choice test asking 4–7 years old children to point out the shape that will come into being when the previously presented two shapes are combined. Figure 3 shows one of the test items of the CMTT.

**FIGURE 4:**
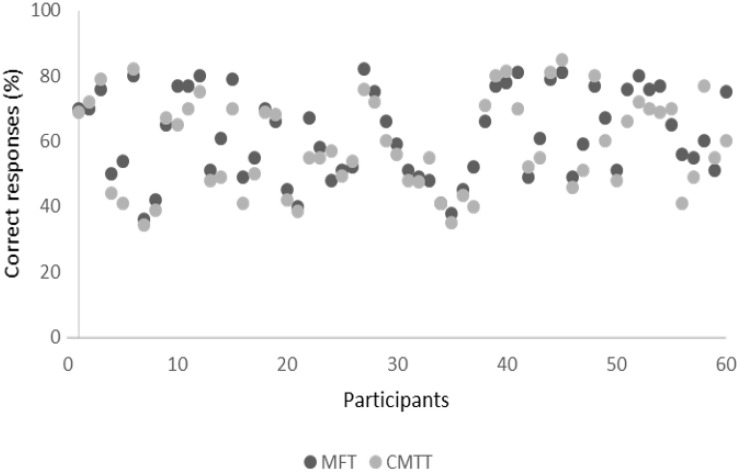
The distribution of the 6 years-children’s spatial ability in each test.

For each test the percentage of correctly solved items related to the total number of items was calculated; the mean of the two scores represented each participant’s spatial abilities. Figure 4 shows the distribution of the 6 years-children’s spatial ability in each test. The correlation of MFT and CMTT was significant with *r* = 0.88.

#### Self-Report of Mental Effort

**FIGURE 5:**
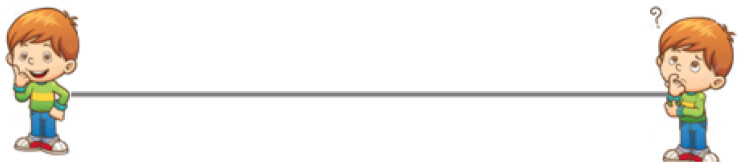
Pictorial illustration of the mental effort scale for children (from very low mental effort – at left – to very high mental effort – at right).

The experimenter explained to the children that he would like to know how they were feeling after the learning phase. In particular, they instructed them to indicate “*How much thinking did you do to complete the task; did you do a lot of thinking or only a little*?” Subsequently, the experimenter presented a picture and said: “*In this picture, the little boy seems to be thinking very hard*.” Then the experimenter pointed to the other picture and said, “*In this picture, he does not seem to be thinking hard at all. How did you feel in the task you just performed*? Finally, the experimenter asked children to place a hash mark between the two pictures (100-mm) and encouraged them to use the full range of the line. Scores were determined by measuring the placement of the hash mark on the 100-mm line. The children were reassured that there were no right or wrong answers. Figure 5 provides a pictorial illustration of the mental effort scale.

#### Motor Recall Performance

Children were asked to accurately recall and execute – in a well-arranged area form the schoolyard – the game sequence. To ensure a smooth running of the situation an individual was instructed to intervene – by providing an oral corrective feedback – each time the children performed a wrong action. For each correct action in the recall test, the participants were assigned one point with a maximum score of 15 points, otherwise, they received zero points.

#### Hesitation Time

This variable represents the time that elapses between the end and the start of a new action made by the participant. It corresponds to the moments of immobility or steps backward (recall of already executed actions).

### Procedure

The session lasted about 30 min, and only one child was tested in each session. First, children completed the spatial ability tests. Afterward, each child was randomly assigned to one experimental condition and was instructed to memorize as precisely as possible the evolution of the game sequence after viewing it one time only. Finally, after the learning task, the computer was switched off, and the post-tests were administered.

### Statistical Analysis

To test the mediating effect of spatial ability, we performed mediation analysis using the pre-specified Model 1 of PROCESS macro (Hayes, 2013). PROCESS application developed by Preacher and Hayes (2008) which is an SPSS procedure (PROCESS, v2.13) that facilitates path analysis and mediation analysis by using ordinary least squares regression (Hayes et al., 2017). An analysis using 5,000 bootstrap samples with 95% confidence levels of the CIs was performed after mean-centering the continuous predictor variables. Three separate moderation analyses were performed in which spatial ability served as moderator variable and recall performance, hesitation time or subjective ratings of cognitive load were used as dependent variables. Significance was accepted for all analyses at the level of *p* ≤ 0.05.

## Results

**FIGURE 6:**
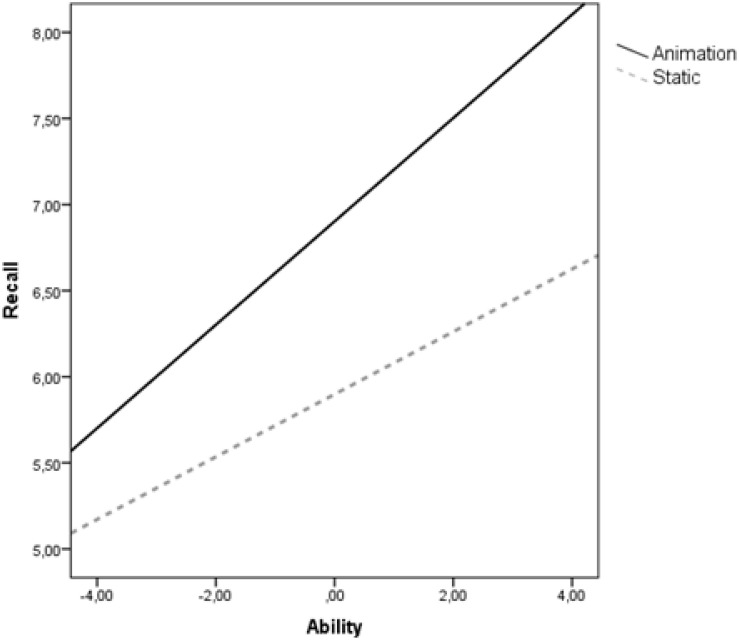
Motor recall performance moderated by spatial ability.

The results for motor recall performance show a significant regression model, *R*^2^ = 0.57, *p* < 0.01. The regression analysis showed significant main effects of both spatial ability [β = 0.419, se(HC4) = 0.094, *p* < 0.001] and condition [β = −1.004, se(HC4) = 0.218, *p* < 0.001] on recall, and an interaction effect between spatial ability and condition [β = −0.088, se(HC4) = 0.06, *p* = 0.04]. Children with high level of spatial ability performed significantly better in the animation condition than in the static condition, while children with low spatial ability achieved the same performance regardless the experimental condition (Figure 6).

The results for cognitive load showed a non-significant regression model, *R*^2^ = 0.078, *p* = 0.137. The regression analysis showed no main effect for spatial ability [β = 0.048, se(HC4) = 0.159, *p* > 0.05], a marginal main effect of condition [β = 0.467, se(HC4) = 0.261, *p* = 0.06], and no interaction of spatial ability and condition [β = −0.060, se(HC4) = 0.093, *p* > 0.05].

**FIGURE 7:**
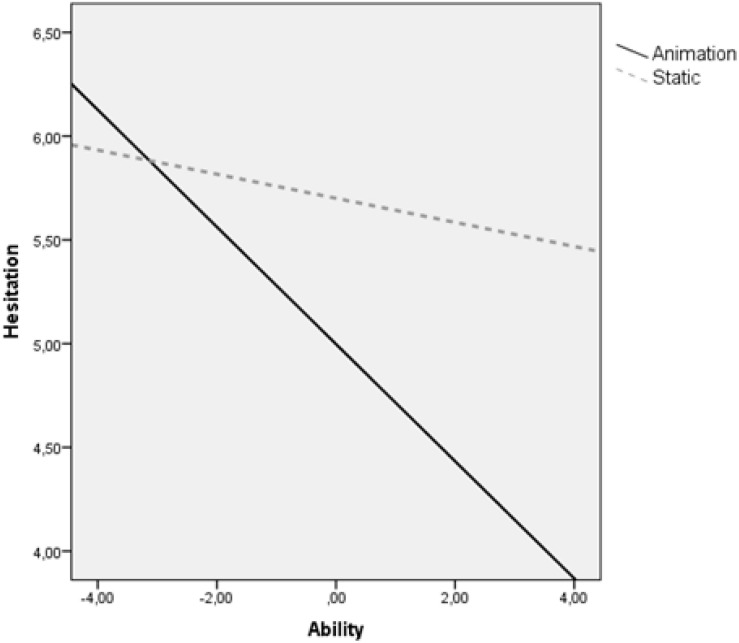
Hesitation time moderated by spatial ability.

The results for hesitation time showed a regression model, *R*^2^ = 0.191, *p* = 0.002, that was significant. The regression analysis showed a significant main effect for spatial ability [β = −0.507, se(HC4) = 0.181, *p* = 0.007], no main effect of condition [β = 0.703, se(HC4) = 0.399, *p* = 0.10], and a marginally significant interaction effect between spatial ability and condition [β = 0.224, se(HC4) = 0.123, *p* = 0.07]. Children with high level of spatial ability reduce their hesitation time in the animation condition compared to the static condition, while children with low spatial ability keep the same hesitation time regardless the type of visualization (Figure 7).

## Discussion

In the current study, a group of primary school children were asked to remember and execute 12 elements of play, shown from static and dynamic 3D visualizations. The moderating role of spatial ability was investigated with respect to motor recall performance, hesitation time and experienced cognitive load.

In line with Hypothesis 1, the results of this study showed that children receiving animations performed better compared to children receiving the static pictures (i.e., they achieved higher motor recall, invested less mental load, and needed less time). This indicates that the explicit presentation of the dynamic aspect of the game such as trajectory and motion helped children in constructing a deeper understanding of the game sequence. Thereby, this dynamic information can directly be read off from the animation, which in turn reduces extraneous cognitive load and incertitude (expressed by the hesitation time before each motor recall). In contrast, with static pictures, this dynamic information needs to be inferred by children via an animation mental process, which is generally assumed to be a more demanding cognitive task than merely perceiving temporal changes (Hegarty et al., 2003). Moreover, previous research have revealed that dynamic visualizations can be more effective form of instruction if they are realistic and involve procedural knowledge (Höffler and Leutner, 2007; Ayres et al., 2009; Wong et al., 2009; Garland and Sanchez, 2013; Castro-Alonso et al., 2015). In this research, we used animations that followed the prescriptions of this earlier research.

Another important finding of this study was the significant interaction found between spatial ability and type of visualization indicating an ability−as−enhancer hypothesis. Children with high spatial ability performed better from animation rather than static pictures (i.e., they achieved higher motor recall, needed less hesitation time and invested the same amount of mental load). It seems that these learners had already developed cognitive capabilities that enabled them to fluently process the fleeting dynamic information without a cognitive overload. However, in the static presentation, they would need to reconcile their cognitive resources with instructional details that were for them redundant and superfluous, which might impose additional extraneous cognitive load and reduce relative general performance. On the other hand, the results revealed that children with low spatial ability do not gain particular benefit from animation (i.e., they achieved the same recall motor score, they invested same amount of mental load and needed the same hesitation time). Because animations change continuously over time, these learners may not be able to process and integrate specific key elements of information that occur within the flow of information (e.g., Lowe, 1999; Rasch and Schnotz, 2009). In contrast, learning from static pictures is self-paced in the sense that learners were allowed as much time as they needed to reinspect a particular information. Therefore, it is likely that interactive animations, which allow children to control the progress of the animation, might be more helpful than animations that play at a fixed rate.

As is the case for all experimental studies, there are some limitations to the generalizability of our results. The limiting variables include the participants used for this research, which included primary school children (in Grade 1); the subject matter area, which focused on motor-procedural learning; and the design of learning materials used, which consisted of a computer-based projection with restricted interactivity. Further research needs to investigate whether our findings can be applied to younger or older children, other subject matter areas, and types of learning materials. Another limitation of the present study is that we did not controlled some moderating variables frequently encountered in animation research (Castro-Alonso et al., 2016), such as the quantity of elements depicted (number bias, i.e., number of images depicted is different in static and animated format), and the visualization format size (size bias, i.e., the animation is larger than the 12 static pictures). Future research could test whether eliminating/minimizing these biases (e.g., by designing all visualizations with the same dimensions) actually influence the learning outcomes. In this study, it was demonstrated that even children with high spatial abilities failed to effectively learn from static pictures. Further research should examine the effect of some external supports considered as helpful in adults (e.g., arrows-indicating motion; Imhof et al., 2013) on children mental animation abilities. Previous studies (e.g., Jarodzka et al., 2010; Mehigan et al., 2011) showed that the way of gazing at the external visualizations is strongly related to individual differences. It would be interesting therefore to employ eye tacking measurements to assess how children with different levels of spatial abilities gaze at animations and static pictures while learning.

## Conclusion

In sum, these results suggest caution in the use of static pictures to convey dynamic information to young children. The static format is considered as the basic visual tool to communicate explicit visual movement may, but this study offers no reason to conclude that static format inherently provides more educational value than animation format. The difficulty to learn from static pictures is noticeable among both low and high spatial abilities children. In the end, casting more light on the way in which children use static and dynamic information will hopefully provide valuable input to teaching, learning, and the design of effective learning materials.

## Data Availability Statement

The raw data supporting the conclusions of this article will be made available by the authors, without undue reservation.

## Ethics Statement

The studies involving human participants were reviewed and approved by the parents were required to consent to the inclusion of their child in the study and provide basic information on the child’s developmental history. The study was conducted according to the Declaration of Helsinki and fully approved the Sfax University Ethics Committee (approval code CPP 0076/2017). Written informed consent to participate in this study was provided by the participants’ legal guardian/next of kin.

## Author Contributions

All authors listed have made a substantial, direct and intellectual contribution to the work, and approved it for publication.

## Conflict of Interest

The authors declare that the research was conducted in the absence of any commercial or financial relationships that could be construed as a potential conflict of interest.
